# The Therapeutic Value of a Becoming Costume

**Published:** 1919-05-03

**Authors:** 


### THE CLOTHING OF LUNATICS.

### The Therapeutic Value of a Becoming Costume.

The Leeds Board of Guardians has protested to the Committee of the West Riding Asylum against the unsuitable clothing of the female patients in that asylum. One of the guardians compared the dress to that of the dame in pantomime, and the lady members of the board spoke of it as "pathetic" and "dreadful."

When some hundreds, it may be more than a thousand, women, many of them utterly demented, incapable of putting on their own clothes, and of faulty habits, are to be clothed at the public expense at a minimum of cost, it is impossible to dress them all tastefully or even becomingly. Quite apart from the underclothing, many of the dresses must be frequently washed, and the process does not tend to preserve either shape or fit. When the dresses return from the laundry in scores, and there are many patients to be supplied, some of whom will not wear the dress for more than a day before it must be washed again, the problem of distributing each dress to the wearer for whom it is most appropriate becomes difficult. The dresses are made in the asylum; the labour of patients is utilised in making them; the cutter has not been trained at Worth's; and the shape and fit necessarily leave a good deal to be desired. All this must be freely admitted in defence of the asylum authorities; but still, there is a good deal that they could do and do not, to improve the dress of the women.

The demented patients and those of offensive habits we may leave out of account. Little can be done for them, and that little they would not appreciate; but, leaving them out of account, much might be done for the others. The clothing is insufficiently varied. The stuff is of the same texture, the colours show very little variety, the pattern, style, and cut of the dress show none at all. This need not be so, and should not be so. It must be so when the dresses are distributed to any one they happen to fit, but this is not necessary.

In every pauper asylum that is reasonably well conducted there are a few women who either make their own dresses or are supplied with them from home, and make a startling and pleasing contrast with the rest. In every large asylum there are certain patients who have made their livings as dressmakers or sempstresses, and are still competent to pursue their trade. In every asylum there are some female patients who, though not professional sempstresses, are quite competent to cut out and make their own clothes, and habitually do so when they are at home. It seems obvious that all these should be permitted and encouraged to make their own dresses out of the asylum material, and that such dresses should be kept for their respective and exclusive use.

Most pauper asylums have now annexes for the accommodation of private patients, in many cases at very small fees, though in many cases it is to be regretted that the visiting committees embark upon a commercial venture, and compete against licensed homes for patients who can afford large fees, and for whom the visitors charge fees out of proportion to the benefits afforded. In those asylums that restrict their accommodation, as it ought to be restricted, to patients who can afford from fifteen or twenty to forty shillings a week, no difficulty whatever is found in supplying to each patient her own proper costume, provided at her own expense or that of her friends. Some of these private annexes are of considerable size, and accommodate large numbers of patients. It is difficult to believe that what is done in the private annexe could not be done in the part of the asylum devoted to paupers.

It may seem, perhaps, that this is an unnecessary fuss to make about a matter so medically unimportant as the becomingness of female costume, but it is not at all unimportant, even medically, in mental disease. In many cases of mental disease a chief difficulty is to restore to the patient self-respect, and what self-respect can be expected of a woman who is dressed hideously in a costume the exact replica of every one that she sees around her? The thing is impossible, and as a therapeutic measure, if for no other reason, every possible endeavour should be made to individualize and render seemly and becoming the costume of the patients in asylums.

